# Cell Division Patterns in the Peristomial Layers of the Moss Genus *Costesia:* Two Hypotheses and a Third Solution

**DOI:** 10.3389/fpls.2020.536862

**Published:** 2020-09-04

**Authors:** Michael S. Ignatov, Ulyana N. Spirina, Maria A. Kolesnikova, Juan Larraín, Elena A. Ignatova

**Affiliations:** ^1^Faculty of Biology, Lomonosov Moscow State University, Moscow, Russia; ^2^Tsitsin Main Botanical Garden, Russian Academy of Sciences, Moscow, Russia; ^3^Faculty of Biology, Tver State University, Tver, Russia; ^4^Instituto de Biología, Pontificia Universidad Católica de Valparaíso, Valparaíso, Chile

**Keywords:** Gigaspermaceae, peristome, bryophytes, haplolepideous, diplolepideous, morphogenesis

## Abstract

The Chilean endemic genus *Costesia* belongs to the Gigaspermaceae, one of the most basal groups of arthrodontous mosses. While none of the species in this family has a peristome, earlier stages of sporophyte development often disclose its basic structure. The study of *Costesia* sporophytes at the early stages of development was conducted to identify possible similarities with *Diphyscium*, the genus sister to Gigaspermaceae plus all other arthrodontous mosses in the moss phylogenetic tree. *Diphyscium* shares a strongly unequal cell division pattern with the Dicranidae. In groups more closely related to *Diphyscium*, as it is the case of *Costesia*, this pattern is not known. Our study of *Costesia* found only irregular presence of slightly unequal cell divisions that may then be considered as a plesiomorphic state in peristomate mosses. The most frequently present pattern revealed in *Costesia* is common with the Polytrichaceae, a more basal moss group with nematodontous peristomes.

## Highlights

We looked for the presence of unequal cell divisions in peristomial layers of *Costesia*, one of the most basal lineages of arthrodontous mosses. Although found at the earliest stages, unequal cell divisions are not stably exhibited throughout the division process. The main developmental pattern in this genus unexpectedly links it to the nematodontous peristome type of the Polytrichaceae.

## Introduction

**Figure 1:**
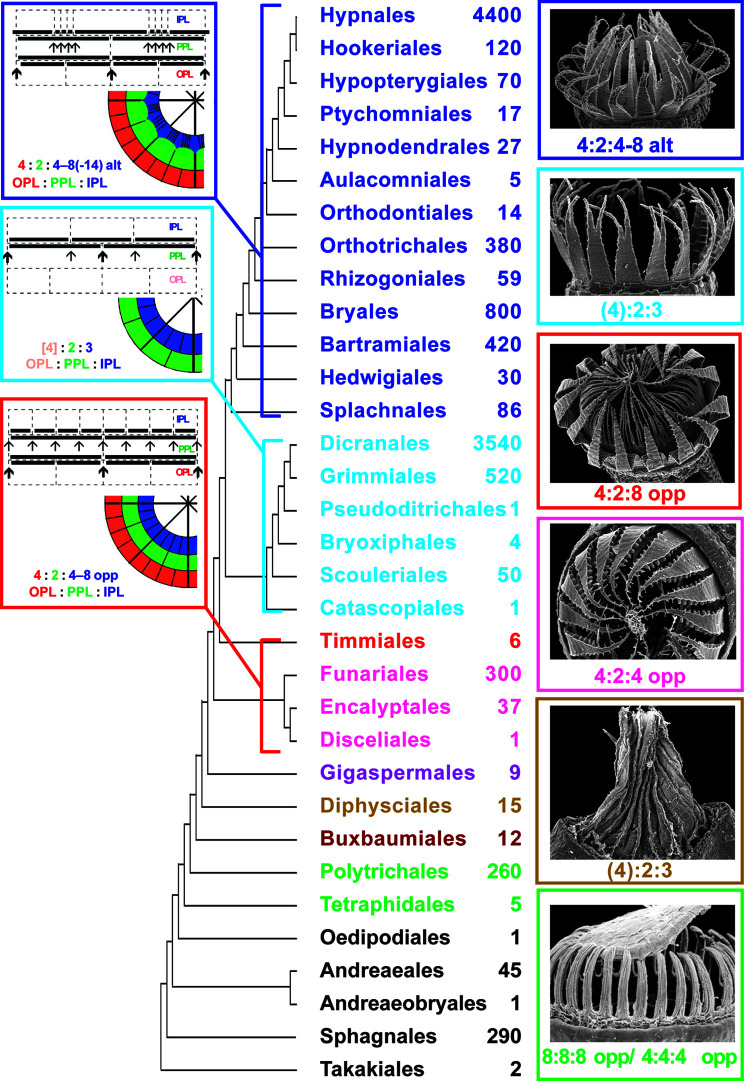
Phylogeny of mosses, following in general Liu et al., 2019, showing the approximate number of species in orders calculated from literature data (e.g., Flora of North America North of Mexico, 2007, 2014) and Tropicos Database (http://legacy.tropicos.org/namesearch.aspx, accesses 15 January 2020). SEM images of one representative with most common structure of completely developed peristome and most common peristomial formula are also given for each lineage of peristome diversification (nematodontous, diplolepideous opposite, haplolepideous, and diplolepideous alternate). Orders of eperistomate mosses are given in black, while orders of peristomate mosses of one subclass (or class) are given in one color: Tetraphidopsida (light green), Polytrichopsida (green), Buxbaumiidae (dark brown), Diphysciidae (brown), Gigaspermidae (purple), Funariidae (magenta), Timmiidae (red), Dicranidae (cyan), Bryidae (blue). Schemes illustrating peristomial formulae of the diplolepideous opposite, haplolepideous, and diplolepideous alternate peristomes are also shown. Characteristic representatives are illustrated with SEM images from Ignatov and Ignatova (2003, 2004), based on writing permission).

The peristome is a structure at the mouth of moss capsules, which enhances the process of spore release by means of hygroscopic movements. The role of the peristome for Bryophyta classification is as important as that of flower and fruit structure for Magnoliophyta. Molecular phylogenetic data in general coincide with the classification based on the peristome structure, defining main subclasses (Newton et al., 2000; Frey and Stech, 2009; Goffinet et al., 2009; Shaw et al., 2011). Recent comprehensive analysis by Liu et al. (2019) summarized molecular data and built a robust phylogenetic tree, where peristomate mosses are found in a grade of seven classes/subclasses and two terminal clades, the subclasses Bryidae (with diplolepideous alternate peristomes) and Dicranidae (with haplolepideous peristomes). These two subclasses include no less than 90% of the contemporary species diversity. Accordingly, the mosses of the grade from Takakiales to Timmiales (**Figure 1**), have much fewer species in the modern flora.

The peristomes of taxa not included in the Bryidae or the Dicranidae are more diverse and not as well studied compared to peristomes of these two terminal clades. Especially enigmatic is the transition from mosses with nematodontous peristomes, formed by entire cells, to mosses with arthrodontous peristomes. The latter are understood in recent literature as a group starting from *Diphyscium* (Shaw et al., 1987; Goffinet et al., 2009), although the distinction between arthrodontous and nematodontous mosses was treated differently. Originally, Mitten (1859) defined neamtodontous peristome as lacking conspicuous transverse cell wall remains, which was followed, among others, by Philibert (1884), translation by Taylor (1962). Fleischer (1904) was the first who grouped Polуtricaceae and Tetraphidaceae together; however in the ‘Fleischer-Brotherus system’ in Die Natürlichen Pflanzenfamilien (Brotherus, 1925) Tetraphidaceae is placed near Bryaceae. Later Dixon (1932) included *Tetraphis* into nematodontous mosses, but together with Buxbaumiales, Diphysciales, Schistostegales, and Calomniales. Until recently, the Polytrichopsida was placed in phylogenetic systems ancestral to Tetraphidopsida (Goffinet et al., 2009). Morphology also supports the position of *Tetraphis* closer to arthrodontous mosses than to the Polytrichaceae (Ligrone and Duckett, 2011), and Shaw and Anderson (1988) concluded that the peristome development in *Tetraphis* is more similar to arthrodontous mosses than to the Polytrichaceae. However, the expanded analyses of Liu et al. (2019) found Tetraphidaceae in a more basal position than Polytrichaceae, albeit with low support. Bell et al. (2020) showed that the Tetraphidaceae is sister to the Polytrichaceae plus all other peristomate mosses in the analysis of plastid data, whereas mitochondrial data places these two families in a clade sister to all other peristomate mosses. In summary, the interrelationships of nematodontous mosses and especially the transition to arthrodontous mosses remains insufficiently understood, being inconsistent in studies of different authors.

The peristome types are characterized by a peristomial formula established by Edwards (1979), that consists of numbers of cells in three (excepting rare exotic cases) peristome-forming layers, so called the outer, primary and inner peristomial layers (OPL, PPL, and IPL), as defined by Blomquist and Robertson (1941). The numbers in the peristomial formulae correspond to the numbers of cells in one-eighth of each layer. Importance and variants of peristomial formulae are only briefly illustrated here in **Figure 1**, as they are discussed in detail in numerous publications (Edwards, 1979; Edwards, 1984; Shaw et al., 1987; Shaw et al., 1989a; Shaw et al., 1989b; Shaw et al., 2011; Ignatov et al., 2015; Ignatov et al., 2018a).

Despite three main peristomial types, the diplolepideous opposite, haplolepideous, and diplolepideous alternate, were found remarkably strictly confined to three main phylogenetic lineages of mosses, Funariidae, Dicranidae and Bryidae (**Figure 1**), the question about the ancestral state remains open. The limited number of peristome development studies precludes the complete understanding of its evolution (Liu et al., 2019). Specifically, the opposite versus alternate arrangement of exostome against endostome elements remains enigmatic (Budke et al., 2007). One of the intriguing points is how *Diphyscium* and Dicranidae, separated by the “diplolepideous opposite” groups in the grade from Takakiales to Timmiales (**Figure 1**) share strongly unequal cell divisions, appearing in a 4:2:3 pattern at the early stage of peristome development.

Searching for an answer to this question, we addressed the Gigaspermaceae which has an intermediate phylogenetic position between *Diphyscium* and all other arthrodontous mosses. The Gigaspermaceae includes five genera, and none of them has a peristome (Goffinet et al., 2009). The numerous peristome development studies done in eperistomate mosses of the genera *Aphanorrhegma, Ephemerum*, and *Physcomitrium* have brought interesting results (Schwartz, 1994). One species of Gigaspermaceae, *Lorentziella imbricata* (Mitt.) Broth., was also studied for sporophyte development by Rushing and Snider (1980), however the strong peristome reduction in that cleistocarpous genus, as well as an undeveloped methodology prior the peristomial formula approach appeared, did not shed enough light on the problem.

Liu et al. (2012) phylogenetic analysis revealed two lineages in the Gigaspermaceae, one with strongly reduced sessile sporophytes including two genera, *Gigaspermum* and *Lorentziella*, and a second clade including *Oedipodiella*, *Chamaebryum*, and *Costesia*, the two latter having elongate setae, i.e., with less reduced sporophytes. *Costesia* was found in the basal position to the two other genera of the second clade, and therefore is the most promising for peristome studies. Two alternative hypotheses were tested: the first one implies that *Costesia* possesses a 4:2:3 pattern, inherited from the Diphysciales, which is the closest ancestral group in the phylogenetic tree (**Figure 1**); an alternative hypothesis was that *Costesia* has opposite cell arrangement in peristomial layers similar to that in diplolepideous opposite groups, the Funariidae, where Gigapermaceae were placed earlier, and which is the closest descendant group in the grade from Takakiales to Timmiales.

## Materials and Methods

### Samples

Plants of *Costesia macrocarpa* (Schimp.) Cuvertino, Miserere & Buffa (= *C. spongiosa* Thér.) with young sporophytes were collected from Lago Peñuelas National Reserve, in Valparaíso Region, central Chile (Chile, Valparaíso Region, Valparaíso Province, National Reserve Lago Peñuelas, 33°10’58”S, 71°29’12”W, 338 m alt., copse of *Quillaja saponaria*, on sandy soil, 24 June 2019, Ignatov, Ignatova & Larraín, s.n., MHA).

Some living plants were subsequently cultivated in the testing chamber MLR32 Sanyo (temperature + 7°С/+ 12°С (night/day), light period 10 h, PPFD - 14μmol • m^-2^ • s^-1^), thus enabling various stages of sporophyte development to be studied.

Additionally, the sections of young sporophytes of *Atrichum undulatum* (Hedw.) P. Beauv. were included for comparison. Specimens were fixed and observed with the same protocols outlined below (voucher: Moscow, Tsitsin Main Botanical Garden, 11 Sept 2019, Ignatov & Spirina s.n., MHA).

### Anatomy Studies

All material collected in the field was fixed shortly after collecting in 2.5% glutaraldehyde in 0.05M PBS. Further steps were performed after several weeks or a few months. Specimens were post-fixed with 1% osmium tetroxide in PBS, pH 6.8, for 6 h. The material was then dehydrated through an ascending ethanol-acetone series to 100% acetone. Next, samples were embedded in Araldite 6005 medium, according to the manufacturer’s protocol. Sections were cut 2 µm thick with glass knives, put on glass slides without mounting medium, stained with 0.01% berberine or in combination with DAPI, and scanned under LSCM Olympus FV-1000 based on Olympus BX61, using 473 nm or combination of 405 and 473 nm lasers. Z-stacks of several scans were usually obtained and are presented here.

We made a complete series of sections 2 µm thick, allowing measure the distance from the section where sporophyte apical cell first appears within the ring of calyptra. Sectioning was stopped when patterning of peristomial layers became irregular. The series show different stages of sporophyte development, as can be assumed from the number of cells in the peristomial layers, especially in IPL, which however do not necessarily correspond to the length and width of sporophytes. Altogether twenty series of transverse sections and sixteen series of longitudinal sections were done, of which eleven series of transverse sections represent specific patterns discussed below (**Supplementary Materials 1**). An additional study of *Atrichum undulatum* was done for comparison after a pattern resembling Polytrichaceae was noticed in *Costesia*. It included serial sections for four young capsules; razor blade sectioning of fresh material was also made in order to confirm that the pattern seen in serial sections can readily be found in other populations.

## Results

**Figure 2:**
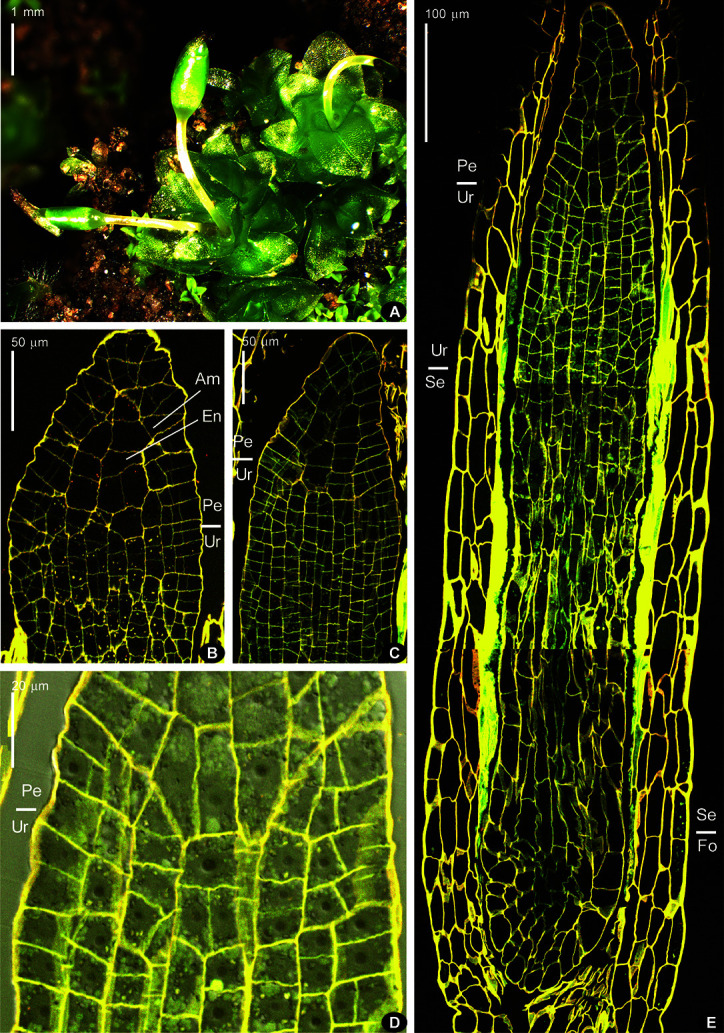
*Costesia macrocarpa* habit (under stereomicroscope) and longitudinal sections (LSCM) of young sporophytes. **(A)** habit of plants with immature capsules, note sporophyte on the far right with strongly curved seta, a not rare case, and two sporophytes in one perichaetium in the center; **(B)** upper part of sporophyte, with peristome zone ca. 80 µm long; (Am), amphithecium, (En), endothecium; **(C)** next stage of sporophyte development, cells in urn zone are arranged in rather regular rows, but still not differentiated into sporogeneous tissue and columella; **(D)** close up of the transition from urn zone (Ur) to peristome zone (Pe), where the direction of cell compartments in amphithecium turns to oblique; **(E)** epigonial stage of sporophyte development, with “peristome zone” (Pe), “urn zone” (Ur), “seta zone” (Se), and “foot zone” (Fo). Bright fluorescence of mucilage is especially conspicuous in the seta zone.

**Figure 3:**
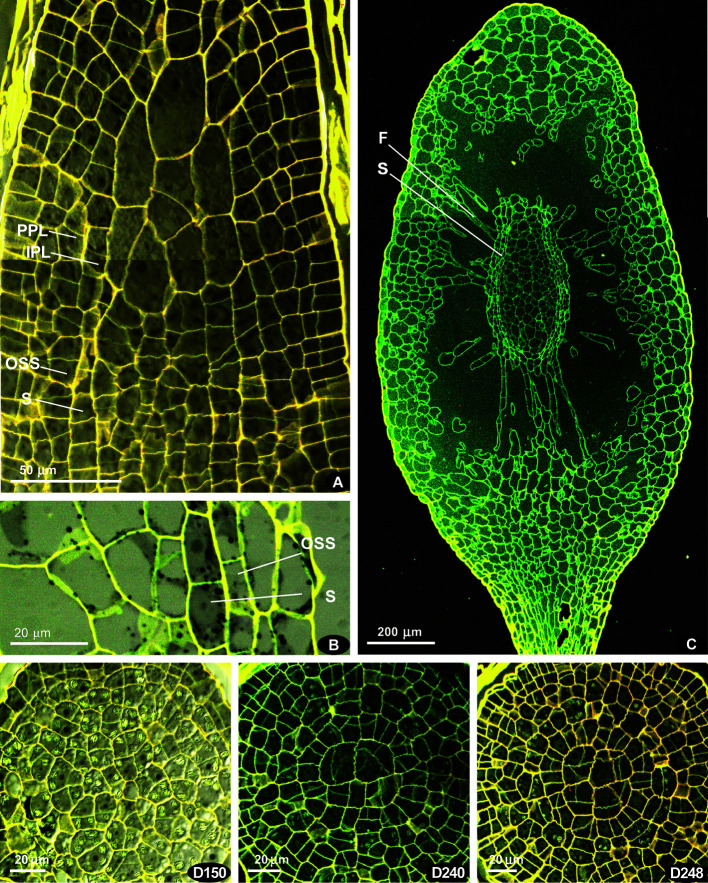
*Costesia macrocarpa*, longitudinal sections, LSCM. **(A)** beginning of differentiation of the sporogeneous layer (S), discernible by quadrate cell shape; note also a relatively regular arrangement of cells in lower part of the peristomial zone; **(B)** spore sac part, with sporogreneous layer contrasted by adding of pseudo-transmission channel of the LSCM; **(C)** capsule, showing the position of spore sac; **(D)** series of transverse sections of capsule of moderately late stage, though earlier than shown in **(C)**; peristomial layers lost patternization at this stage except the innermost ring of longitudinally transverse cells (D248); numbers indicate the distance from the sporophyte apex in μm.

The longitudinal sections represent plants at stages from a whole sporophyte length of 0.4 mm (**Figure 2B**) to a length of 1.7 mm for capsule only (**Figure 3C**), when the sporogeneous layer is apparently differentiated. In longitudinal sections of young sporophytes, the area of enlarged cells is seen right below the apex, corresponding to the endothecium at the level where peristome is formed in most moss species (**Figure 2**). It is formed of large cells 20–30 µm wide (**Figure 2B**), without or with very few cell divisions in them. Cells below this zone are smaller because much more divisions took place. The amphithecium compartments are perpendicular to capsule wall in the lower part of this “peristome zone”, and the more distal, the more oblique they are. Cells of the “urn zone” at earlier stages (amphithecium 1–3 cells thick, width of sporophyte at annulus level 110–130 µm, **Figures 2B, C, E**) are arranged in longitudinal rows without apparent differentiation. Later (amphithecium 4–5 cells thick, width of sporophyte at annulus level 160 µm, **Figure 3A**) cells in the middle of the “urn zone” become more variable in shape, while rows connected to lower cells of endothecium in “peristome zone” are more or less apparently differentiated. Their relative regularity and position correspond to their further differentiation into sporogeneous tissue.

At early stages, when the whole sporophyte is ca. 800 µm long, the “peristome zone”, “urn zone”, “seta zone”, and “foot zone” occupy ca. 150, 250, 250, and 150 µm respectively (**Figure 2E**). The “seta zone” is composed of strongly elongate cells, while the foot includes numerous short cells with thin walls, becoming especially wavy due to fixation (**Figure 2E**).

**Figure 4:**
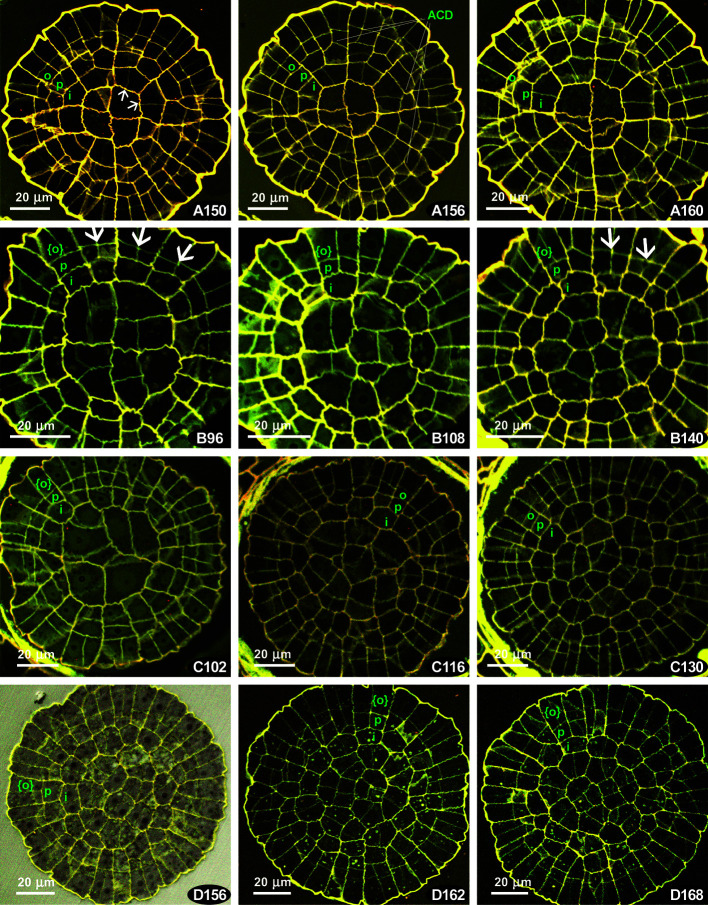
*Costesia macrocarpa*, transverse sections of young capsules, LSCM. **(A–D)** four series of transverse sections; numbers indicate the distance from the sporophyte apex in μm. Small arrows point to anticlinal cell walls resulted in offset divisions in IPL; large arrows point to additional divisions in PPL, resulting in {4}:4:2 formula; ACD, anticlinal curved divisions. The OPL, PPL, and IPL layers are denoted by o, p. i, and in the case of three cells thick amphithecium the outermost layer is marked as {o}.

Later on, cells of the ‘peristome zone’ additionally divide and enlarge, forming parenchymal cells beneath the operculum. The ‘urn zone’ is five times enlarged both longitudinally and transversally, and the spore sac is separated from the capsule wall, and hangs on filaments (**Figure 3C**). In the spore sac, the sporogeneous layer is differentiated (**Figure 3B**) by darker color of less vacuolated cells, more regular in shape compared to the tissue of the massive columella. The outer spore sac layer continues in the peristomial zone in the IPL layer (**Figure 3A**). At the stage shown in **Figures 3C, D240, D248**, the regular patterns in the “peristome zone” are already lost. Cells above the urn are irregularly arranged, except only at the level of the uppermost ‘urn zone’, where cells around endothecium form a conspicuous ring of radially elongate cells, at places approaching four per 1/8 sector. At this stage operculum tissue is much expanded (**Figure 3** D150, cf. 3C). However, at the earlier stage (**Figure 3A**), cells are regular enough to see patterns characteristic for peristomial layers in transverse sections (**Figures 4**).

These regular cell patterns are apparent only in a limited interval of 10–20 μm, rarely up to ca. 40 μm, corresponding to levels shortly above the urn top (**Figure 3A**). They are not distinct in all the sectors of the circumference (cf. **Figure 4** and **Supplementary Materials 1**) and not in all series of sections, being still not expressed in the youngest one, where amphithecium is only two-layered, and the oldest, where cells of peristomial layer lose their regular arrangement (**Figure 3D**248). However, the peristomial formula observed in most octants in capsules on the stages where the cell patterns are most evident (**Figure 4**) deserves to be discussed.

Four series of transverse sections are shown in **Figure 4**. Here we show different sporophyte developmental stages, which however do not necessarily correspond to the length and width of the sporophytes, and therefore we arrange them by the number of cells, especially in IPL

The series of the youngest sporophytes have too irregular cell arrangements, thus not relevant for the discussion of peristomial layers. The sections with apparent patterning were usually quite a few in *Costesia* compared to other arthrodontous mosses, and the younger the capsule was the fewer were the sections useful for the analysis.

A relatively young sporophyte, putatively comparable with that shown in **Figure 2B** is presented in **Figure 4A**. Peristomial formulae in **Figure 4** A150 are 2:2:1, 4:2:1, and 4:2:2, with anticlinal cell walls in IPL strongly offset (small arrows) against anticlinal cell walls in PPL, thus representing a ‘haplolepideous pattern’. The same pattern is seen in the sections at the next 10 µm. Noteworthy is the presence of anticlinal-curved divisions that cut off PPL cells, and sometimes also in IPL cells, in an unusual way (cf. **Figure 4** A156).

The series B comprises a {4}:4:2 pattern, where two cells of the octant belong to IPL, and four cell to the PPL, while OPL is still undifferentiated. Therefore the cells outwards the PPL, which are outermost amphithecial and epidermal cells at the same time at this stage are shown in braces. The {4}:4:2 pattern appeared in this series since the beginning of the PPL differentiation, cells outwards IPL remain undivided in some octants (i.e., PPL *sensu stricto* is still lacking, **Figure 4** B96), to the level where this {4}:4:2 formula is seen in the majority of the 1/16 sectors (**Figure 4** B140). Thus, the {4}:4:2 formula occurs throughout 44 µm interval, being clearly expressed in between 96 µm and 140 µm from capsule apex in all sections (e.g., **Figure 4** B108).

In the other series were {4}:4:2 pattern occurs, it is expressed usually in 10 to 20 µm intervals at the transition from peristomial zone to urn zone, which is apparent as cells in the middle of the transverse section are smaller and lacking quadrant arrangement (cf. **Figure 4** B96 and C102 with B140 and C130).

**Figure 5:**
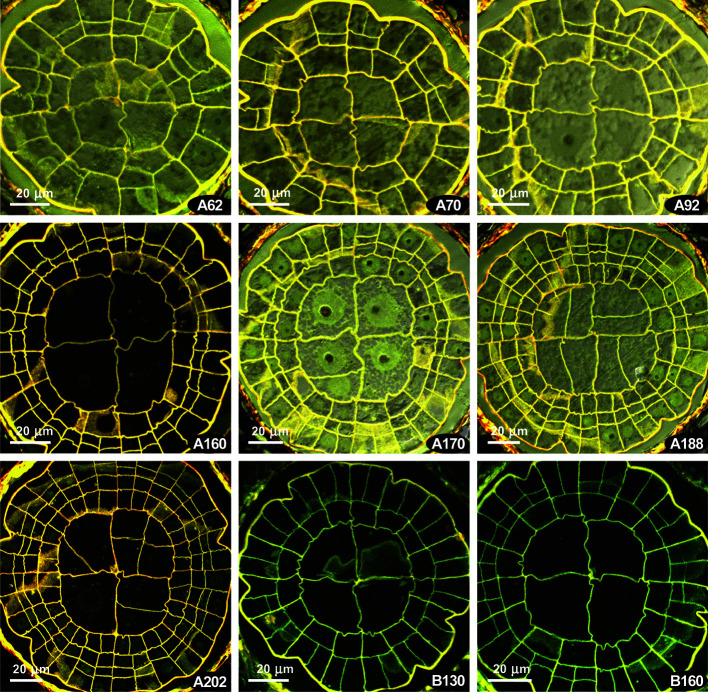
*Atrichum undulatum*, transverse sections of young capsules, LSCM. **(A, B)** two series of transverse sections; numbers indicate the distance from the sporophyte apex in μm.

The presence of the {4}:4:2 pattern characteristic for the Polytrichaceae (Wenderoth, 1931) forced us to compare *Costesia* with the Polytrichaceae, so we studied sporophytes at early stages of development in *Atrichum undulatum*. There is also a variation between capsules, and two variants (out of six studied) are shown in **Figure 5**. Unexpectedly, in some series at the early stages, but where the amphithecium is already three-layered, a number of 2:2 patterns with slight but distinct offset was observed (**Figure 5** A62, A70), although in other series this pattern is lacking and all the divisions were fairly regular, despite sporophytes superficially looked almost identical (**Figure 5B**). Later the formula {4}:4:2 appeared, and then cells in IPL divided more or less precisely aligned to PPL cells, resulting in a {4}:4:4 and later a 4:4:4 (cf. **Figure 5** A170, A188, A202), and 8:8:8 (Wenderoth, 1931). However, in all four series of sections and additional studies of fresh material of *Atrichum* we observed the {4}:4:2 formula along a considerable distance at early stages of peristome development (**Figure 5** and **Supplementary Materials 1**).

## Discussion

The comparison of complete series of the transverse sections with longitudinal sections (**Figures 2** and **3**) ensures that the regular patterns illustrated in **Figure 4** correspond to the level of the proximal part of the “peristomial zone”, thus the discussion on the peristomial formula for *Costesia* should be as important as it is for other eperistomate mosses, like *Physcomitrium* and *Aphanorhegma* (Schwartz, 1994).

The obtained observation revealed two patterns in *Costesia* worth discussing: (1) asymmetric divisions occur in IPL in most distal part of the amphithecium (cf. **Figure 4**, Table in **Supplementary Materials 1**), and (2) number of cells in PPL twice as many as in IPL.

Asymmetric cell divisions in IPL are typically associated with a haplolepideous pattern type. Some strongly asymmetric divisions were found in *Costesia*, but they are irregular and occur in a limited distal part of the sporophyte (**Supplementary Materials 1**). Such irregular asymmetric divisions were also observed in *Atrichum*, but only in a short interval below the sporophyte apex (**Supplementary Materials 1**).

Budke et al. (2007) suggested to separate (and differently score) slightly offset and strongly offset divisions in peristomial layers. These authors came to the conclusion that strong offset anticlinal divisions in the IPL occur in *Diphyscium* and in the majority of the studied haplolepideous mosses, whereas in other groups these divisions are less prominent. The published illustration shows that the slightly offset divisions occur in such basal lineages as *Tetraphis* (Shaw and Anderson, 1988) and *Oedipodium* (Shimamura and Deguchi, 2008), and at early stages of sporophyte development in some representatives of the “diplolepideous opposite” group, e.g., *Physcomitrium* (Schwartz, 1994), and in “diplolepideous alternate” mosses (Shaw et al., 1989a). Taking these points into consideration, the presence of offset divisions in few octants at the earlier stage of development in *Costesia* (**Figure 4**) and even *Atrichum* (**Figure 5**) may not be a significant phylogenetic marker.

**Figure 6:**
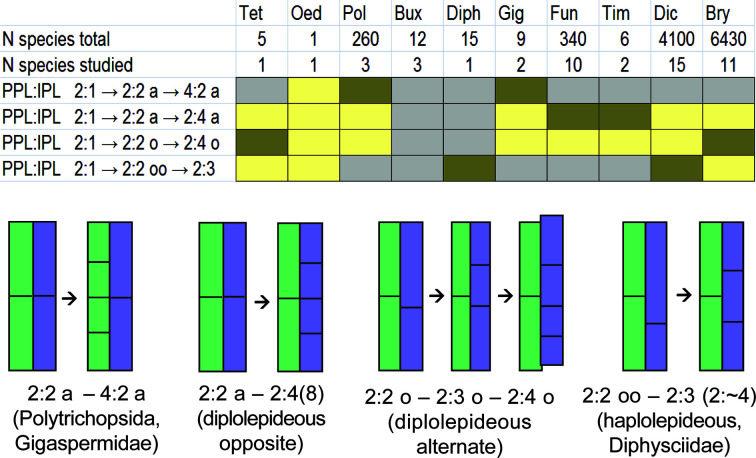
Distribution of aligned, slightly offset and strongly offset positions of the anticlinal cell wall in IPL against corresponding cell wall in PPL at the critical stage of transition from peristomial formula x:2:1 to x:2:2 among classes, subclasses and some orders of peristomate mosses based on literature data combined in **Supplementary Materials 2** and on our observations. Letter ‘a’ indicates aligned position of anticlinal cell walls in IPL against anticlinal cell walls in PPL, letter ‘o’ denotes a slightly offset position (5%–32%), while “oo” means a strongly offset position (>33%). Oed – Oedipodiopsida; Tet – Tetraphidopsida; Pol – Polytrichopsida; Bux – Buxbaumiidae; Diph – Diphysciidae; Gig – Gigaspermidae; Fun – Funariidae; Tim – Timmiidae; Dic – Dicranidae; Bry – Bryidae. Number of species in these taxa and number of studied species is provided according to **Supplementary Materials 2**. Colors indicate the frequency of occurrence of these characters in a particular group of species: brown – frequent; yellow – rare; grey – not observed; Buxbaumiidae are not referred to any of these cases as their formulae are indefinite (Ignatov et al., 2018b). Schemes below show the position of anticlinal cell walls in PPL (green) and IPL (blue) at the stage of transition from 2:1 to 2:2 (left) which is critical for the subsequent peristome development, and the resulting arrangement of cells in these two peristomial layers (right) in main lineages of peristome diversification. Frequencies are integral from the variation within transverse section and among species, the latter is shown in **Supplementary Materials 2**.

In **Figure 6**, the distribution of various cell divisions is summarized, allowing for the conclusion that the least definite case of slightly offset divisions occurs in most groups in certain taxa, or at certain stages of development (based on published data, see **Supplementary Materials 2**). The developmental patterns based on strictly determined unequal cell divisions occur in Dicranidae and Bryidae, two groups that include no less than 85% of species of the world moss flora. Distinctly aligned cell divisions are characteristic of the arthrodontous Funariales, Encalyptales, Disceliales, and Timmiales, which include less than 3% of species of the known moss diversity. The Funariales represent the largest of the latter orders, and the majority of its species have no peristome or a strongly reduced one. Aligned cell divisions are also a characteristic of nematodontous Polytrichales, to some of which *Costesia* appeared to be most similar in having {4}:4:2 pattern at early stages of development.

The presence of the {4}:4:2 pattern in *Costesia* was noteworthy. No other arthrodontous mosses have such regular presence of additional cell divisions in PPL. Species with endostome adherent to the exostome, e.g., *Catoscopium* (Ignatov et al., 2015), *Encalypta* (Ignatov et al., 2018a), and *Splachnum* (Schwartz, 1994) may have such divisions in PPL, but they are rather rare and commence only at the latest stages of development. Therefore *Costesia* is similar in the presence of this pattern to the family Polytrichaceae, which might look unexpected. However there is another moss where this pattern can also be discerned: this is *Lorentziella*, another genus of Gigaspermaceae. Its sporophyte development was carefully studied by Rushing and Snider (1980), and despite their description did not specifically address this pattern, it is clear from the detailed and carefully prepared illustrations. The early stages of sporophyte development in *Lorentziella* are not specific, being common for all bryophytes (Shaw and Anderson, 1988), whereas at the later stages the pattern {4}:4:2 in “peristomial” layers (four cells in PPL, two in IPL) are seen in several octants. Interestingly, this pattern appears simultaneously with the differentiated layer called by Rushing and Snider (1980) as “endothecium 1”. It differs in a somewhat darker color and indicates the stage (in time)/or level (in space) where the sporogeneous tissue starts differentiation. It can be assumed that this level and stage correspond to what we see in *Costesia* at the level of annulus.

The presence of a {4}:4:2 pattern in *Lorentziella* additionally ensures us that the same pattern in *Costesia* is not occasional and may be discovered in other genera of Gigaspermaceae. How much it can be interpreted in favor of a relationship to Polytrichaceae is a question without answer: no attempts were made to compare these groups previously, because they are drastically different morphologically. The only similarity that is worth mentioning, is the robust capability to form underground rhizomes in both of these families. Although, this type of growth occurs in a number of groups of subclass Bryidae as well. If this coincidence is not simply occasional, it could be understood only after further studies.

## Data Availability Statement

The datasets generated for this study are available on request to the corresponding author.

## Author Contributions

General plan (MI), field work organization (JL). Field work conducting (JL, MI, EI), plant fixation and embedding (US), anatomy study (MK, US), microscopy (US, MK, MI), manuscript preparation (MI, EI, JL).

## Funding

Russia Foundation for Basic Research, 19-04-00976 and MBG Institutional research project 18-118021490111-5.

## Conflict of Interest

The authors declare that the research was conducted in the absence of any commercial or financial relationships that could be construed as a potential conflict of interest.
