# Supplementary material for: Cell Division Patterns in the Peristomial Layers of the Moss Genus Costesia: Two Hypotheses and a Third Solution
Source: Front Plant Sci. 2020 Sep 4;11:536862. doi: 10.3389/fpls.2020.536862 (PMC7498844; doi:10.3389/fpls.2020.536862)
Supplement: Supplementary file 1 [file DataSheet_1.doc]

Supplementary materials 1

Observations on the peristome transverse sections of *Costesia macrocarpa* and *Atrichum undulatum*.

In *Costesia macrocarpa* transverse sections were done on 20 sporophytes, totaling 2220 sections, 2 μm thick each. In the table below, the intervals are given in microns.

Each slide glass had 40 sections. The study focused on the intervals where a pattern was evident (see tables below).

Five sporophytes were too old, i.e. at the late stage (cf. Fig. 3D), already without a visible pattern of cells in peristomial layers.

Two sporophytes were too young, and their amphithecium was two-layered up to the urn level. The other two sporophytes had too irregular cell patterns to be scored.

1. *Costesia macrocarpa*

Series of transverse sections, indicating intervals where 4:4:2 or {4}:4:2 patterns occur. In 11 series a 4:4:2 or {4}:4:2 pattern was seen in 4-7 of 16 sectors of the peristomial circumference, and among them, this pattern was observed in six series (marked by !) in eight or more of 16 sectors of the circumference (cf. Fig. 4B, C, D).

| Series # | total length of studied part of sporophyte, μm | interval where 4:4:2 or {4}:4:2 patterns: (appear–) prevail in more than half octants, μm |
| --- | --- | --- |
| 1 | 160 | young |
| 2 | 240 | 120-140 |
| 3 | 240 | 146-156 |
| 4 | 160 | irregular |
| 6 | 240 | 156-168! |
| 7 | 160 | 158 |
| 8 | 240 | 152-160! |
| 10 | 160 | 128-144! |
| 11 | 240 | old |
| 12 | 240 | old |
| 14 | 400 | old |
| 16 | 240 | irregular |
| 17 | 240 | 170-186 |
| 19 | 240 | 148-156 |
| 20 | 240 | old |
| 24 | 120 | 96-140! |
| 26 | 280 | 150-166! |
| 27 | 160 | young |
| 35 | 240 | 102-130! |
| 36 | 320 | old |

2. *Atrichum undulatum*

Series of transverse sections, indicating intervals where 4:4:2 or {4}:4:2 patterns occur. (cf. Fig. 5).

| Series # | total length of studied part of sporophyte, μm | interval where 4:4:2 or {4}:4:2 pattern: (appear–) prevail |
| --- | --- | --- |
| 1 | 480 | (130-)190-250 |
| 2 | 320 | (~120)160-200 |
| 6 | 400 | (220-)230-280 |
| 7 | 400 | (150-)170-250 |

Additionally, young sporophytes from a population of *Atrichum undulatum* were cut with a razor blade at the upper part of capsules searching for a 4:4:2 or {4}:4:2 pattern. After the immediate finding of this pattern in two sporophytes, the search was canceled, as it became clear that it is always present in all the sporophytes. A picture in Meyer (1922) supports this conclusion as well.

Meyer, K. I. (1922). Historie de dévolopment du sporogone de Catharinea undulata *J. Soc. Bot. Russ*. 7, 109–123.
