# Supplementary material for: Cell Division Patterns in the Peristomial Layers of the Moss Genus Costesia: Two Hypotheses and a Third Solution
Source: Front Plant Sci. 2020 Sep 4;11:536862. doi: 10.3389/fpls.2020.536862 (PMC7498844; doi:10.3389/fpls.2020.536862)
Supplement: Supplementary file 2 [file DataSheet_2.doc]

Supplementary materials 2

Sources of information that is summarized in Fig. 6 (which is shown also here)

|  |  | Tet | Oed | Pol | Bux | Diph | Gig | Fun | Tim | Dic | Bry |
| --- | --- | --- | --- | --- | --- | --- | --- | --- | --- | --- | --- |
|  | N species total | 5 | 1 | 260 | 12 | 15 | 9 | 340 | 6 | 4100 | 6430 |
|  | N species studied | 1 | 1 | 3 | 3 | 1 | 2 | 10 | 2 | 15 | 11 |
| AA | PPL:IPL 2:1 → 2:2 a → 4:2 a |  |  |  |  |  |  |  |  |  |  |
| A | PPL:IPL 2:1 → 2:2 a → 2:4 a |  |  |  |  |  |  |  |  |  |  |
| O | PPL:IPL 2:1 → 2:2 o → 2:4 o |  |  |  |  |  |  |  |  |  |  |
| OO | PPL:IPL 2:1 → 2:2 oo → 2:3 |  |  |  |  |  |  |  |  |  |  |

List of species studied for the peristome development, with corresponding reference and conclusion from illustrations and descriptions on the presence of aligned / offset divisions in the innermost amphithecium layer against periclinal cell wall in the second amphithecium layer from endothecium.

In columns: 0= never, 1= occasional, 2= mostly.

Letters in columns (OO, O, A, AA) correspond to the peristomial formulae indicated in the table above.

| Taxa | Reference | Cell division (cf. Fig 6) | | | |
| --- | --- | --- | --- | --- | --- |
|  |  | OO | O | A | AA |
| Tetraphidopsida |  |  |  |  |  |
| *Tetraphis pellucida* | Shaw & Anderson, 1988 | 0 | 2 | 2 | 0 |
|  |  |  |  |  |  |
| Oedipodiopsida |  |  |  |  |  |
| *Oedipodium griffithianum* | Shimamura & Deguchi, 2008 | 1 | 1 | 1 | 1 |
|  |  |  |  |  |  |
| Polytrichipsida |  |  |  |  |  |
| *Polytrichum commune* | Wijk, 1929 | 0 | 0 | 0 | 2 |
| *Polytrichum juniperinum* | Wenderoth, 1931 | 0 | 0 | 0 | 2 |
| *Atrichum undulatum* | Meyer, 1922; present study | 0 | 1 | 1 | 2 |
|  |  |  |  |  |  |
| Buxbaumiidae |  |  |  |  |  |
| *Buxbaumia aphylla* | Ignatov et al., 2018b | 0 | 0 | 0 | 0 |
| *Buxbaumia minakatae* | Ignatov et al., 2018b | 0 | 0 | 0 | 0 |
| *Buxbaumia viridis* | Ignatov et al., 2018b | 0 | 0 | 0 | 0 |
|  |  |  |  |  |  |
| Diphysciidae |  |  |  |  |  |
| *Diphyscium foliosum* | Shaw et al., 1987 | 2 | 0 | 0 | 0 |
|  |  |  |  |  |  |
| Gigaspermidae |  |  |  |  |  |
| *Costesia macrocarpa* | present study |  | 1 | 1 | 2 |
| *Lorentziella imbricata* | Rushing & Snider, 1980 | 0 | 0 | 1 | 1 |
|  |  |  |  |  |  |
| Funariidae |  |  |  |  |  |
| *Aphanorrhegma serratum* | Schwartz, 1994 | 0 | 1 | 2 | 0 |
| *Discelium nudum* | Shaw & Allen, 1985; Ignatov et al., in prep. | 0 | 1 | 2 | 0 |
| *Encalypta longicolla* | Ignatov et al., 2018a | 0 | 0 | 2 | 0 |
| *Encalypta procera* | Ignatov et al., 2018a | 0 | 0 | 2 | 0 |
| *Encalypta rhaptocarpa* | Ignatov et al., 2018a | 0 | 0 | 2 | 0 |
| *Encalypta vulgaris* | Ignatov et al., 2018a | 0 | 0 | 2 | 0 |
| *Funaria arctica* | Ignatov et al., 2018a | 0 | 0 | 2 | 0 |
| *Funaria flavicans* | Schwartz, 1994 | 0 | 0 | 2 | 0 |
| *Funaria hygrometrica* | Shaw et al., 1989a; Schwartz, 1994 | 0 | 0 | 2 | 0 |
| *Physcomitrium pyriforme* | Schwartz, 1994 | 0 | 1 | 2 | 0 |
|  |  |  |  |  |  |
| Timmiidae |  |  |  |  |  |
| *Timmia bavarica* | Ignatov et al., 2018a | 0 | 1 | 2 | 0 |
| *Timmia megapolitana* | Budke et al., 2007 | 0 | 0 | 2 | 0 |
|  |  |  |  |  |  |
| Dicranidae |  |  |  |  |  |
| *Archidium donnellii* | Snider, 1975 | 0 | 0 | 0 | 0 |
| *Catoscopium nigritum* | Ignatov at al., 2015 | 2 | 2 | 0 | 0 |
| *Ceratodon purpureus* | Evans & Hooker, 1913 | 2 | 0 | 0 | 0 |
| *Dicranum condensatum* | Shaw et al., 1989b | 2 | 0 | 0 | 0 |
| *Dicranum japonicum* | Saito, 1956 | 2 | 0 | 0 | 0 |
| *Dicranum rhabdocarpum* | Shaw et al., 1989b | 2 | 0 | 0 | 0 |
| *Dicranum scoparium* | Shaw et al., 1989b | 2 | 0 | 0 | 0 |
| *Ditrichum cylindricum* | Shaw et al., 1989b | 2 | 0 | 0 | 0 |
| *Ditrichum lineare* | Shaw et al., 1989b | 2 | 0 | 0 | 0 |
| *Ditrichum pallidum* | Shaw et al., 1989b | 2 | 0 | 0 | 0 |
| *Ephemerum serratum* | Schwartz, 1994 | 0 | 2 | 1 | 0 |
| *Fissidens limbatus* | Mueller, 1973 | 3 | 0 | 0 | 0 |
| *Glyphomitrium humillimum* | Estébanez et al., 2006 | 0 | 2 | 0 | 0 |
| *Grimmia pulvinata* | Kienitz-Gerloff, 1878 | 2 | 0 | 0 | 0 |
| *Trematodon longicollis* | Shaw et al., 1989b | 2 | 0 | 0 | 0 |
|  |  |  |  |  |  |
| Bryidae |  |  |  |  |  |
| *Aulacomnium heterosichum* | Blomquist & Robertson, 1941 | 1 | 2 | 0 | 0 |
| *Bartramia crispula* | Saito & Shimoze, 1955 | 1 | 2 | 0 | 0 |
| *Bryum bicolor* | Shaw et al., 1989a | 1 | 2 | 0 | 0 |
| *Bryum pseudocapillare* | Shaw et al., 1989a | 1 | 2 | 0 | 0 |
| *Podperaea krylowii* | Ignatov et al., 2015 | 1 | 2 | 0 | 0 |
| *Schlotheimia rugifolia* | Goffinet et al., 1999 | 1 | 2 | 0 | 0 |
| *Splachnum ampullaceum* | Schwartz, 1994 | 0 | 1 | 2 | 0 |
| *Tetraplodon mnioides* | Schwartz, 1994 | 0 | 1 | 2 | 0 |
| *Ulota crispa* | Goffinet et al., 1999 | 1 | 2 | 0 | 0 |
| *Ulota hutchinsiae* | Goffinet et al., 1999 | 1 | 2 | 0 | 0 |
|  |  |  |  |  |  |

Blomquist, H. L., and Robertson, L. L. (1941). The development of the peristome in *Aulacomnium heterostichum. Bull. Torrey Bot. Club* 65, 569–584.

Budke, J. M., Jones, C. S., and Goffinet, B. (2007). Development of the enigmatic peristome of *Timmia megapolitana* (Timmiaceae; Bryophyta). *Amer. J. Bot.* 94, 460–467.

Estébanez, B., Yamaguchi, T., and Deguchi, H. (2006). The development of an unusual haplolepideous peristome type: *Glyphomitrium humillimum*. *J. Hattori Bot. Lab.* 100, 77–87.

Evans, A. W., and Hooker Jr., H. D. (1913). Development of the peristome in *Ceratodon purpureus*. *Bull. Torrey Bot. Club* 40, 97–109.

Goffinet, B., Shaw, A. J., Anderson, L. E., and Mishler, B. D. (1999). Peristome development in mosses in relation to systematics and evolution. V. Diplolepideae: Orthotrichaceae. *Bryologist* 102, 581–594.

Ignatov, M. S., Spirina, U. N., Ignatova, E. A., Krug, M., and Quandt, D. (2015). On the systematic position of the moss genus *Catoscopium* with a new approach to the peristome reduction study. *Arctoa* 24, 389–415.

Ignatov, M. S., Spirina, U. N., Kolesnikova, M. A., Ashikhmina, D. A., Ignatova, E.A., and Polevova, S. V. (2018a). Peristome development pattern in *Encalypta* poses a problem: what is the primary peristomial layer in mosses? *Arctoa* 27(1), 1–17.

Ignatov, M. S., Spirina, U. N., Kolesnikova, M. A., Volosnova, L. F., Polevova, S. V., and Ignatova, E. A. (2018b). *Buxbaumia*: a moss peristome without a peristomial formula. *Arctoa* 27(2), 172–202.

Kienitz-Gerloff, F. (1878). Untersuchungen uber die Entwicklungsgeschichte der Laubmooskapsel und die Embryoentwicklung einiger Polypodiaceen. Bot. *Zeitung* 36, 33–64.

Meyer, K. I. (1922). Historie de dévolopment du sporogone de *Catharinea undulata*. *J. Soc. Bot. Russ*. 7, 109–123.

Mueller, D. M. J. (1973). The peristome of *Fissidens limbatus* Sullivant*.* *Univ. Calif. Publ. Bot.* 63, 1–34.

Rushing, A. E., and Snider, J. A. (1980). Observations on sporophyte development in *Lorentziella imbricata* (Mitt.) Broth. *J. Hattori Bot. Lab.* 47, 35–44.

Saito, S. (1956). Studies on the development of the peristome in Musci II. On the peristome in *Dicranum japonicum* Mitt. *Bot. Mag., Tokyo* 69(812), 53–59.

Saito, S., and Shimoze, S. (1955). Studies on the development of the peristome in Musci I. On the peristome in *Bartramia crispula* Schimp. *Bot. Mag., Tokyo* 68(800), 55–60.

Schwartz, O. M. (1994). The development of the peristome-forming layers in the Funariaceae. *Int. J. Pl. Sci.* 155, 640–657.

Shaw, A. J., and Allen, B. H. (1985). Anatomy and morphology of the peristome in *Discelium nudum* (Musci: Disceliaceae). *Bryologist* 88, 263–267.

Shaw, A. J. and Anderson, L. E. (1988). Peristome development in mosses in relation to systematics and evolution II. *Tetraphis pellucida* (Tetraphidaceae). *Amer. J. Bot.* 75, 1019–1032.

Shaw, A. J., Anderson, L. E., and Mishler, B. D. (1987). Peristome development in mosses in relation to systematics and evolution 1. *Diphyscium foliosum* (Diphysciaceae). *Mem. New York Bot. Gard.* 45, 55–70.

Shaw, A. J., Anderson, L. E. and Mishler, B. D. (1989a). Peristome development in mosses in relation to systematics and evolution. III. *Funaria hygrometrica, Bryum pseudocapillare,* and *B. bicolor. Syst. Bot.* 14: 24–36.

Shaw, A. J., Mishler, B. D. and Anderson, L. E. (1989b). Peristome development in mosses in relation to systematics and evolution. IV. Haplolepideae: Ditrichaceae and Dicranaceae. *The Bryologist* 92, 314–325.

Shimamura, M., and Deguchi, H. (2008). “Sporophyte anatomy of *Oedipodium griffithianum* (Oedipodiaceae)”, in *Bryology in the New Millennium.* Eds. H. Mohamed, B. B. Baki, A. Nasrulhaq-Boyce, et al. (Institute of Biological Sciences, University of Malaya, Kuala Lumpur, Malaysia), 319–325.

Snider, J. A. (1975). Sporophyte development in the genus *Archidium*. *J. Hattori Bot. Lab.* 39, 85–104.

Wenderoth, H. (1931). Beiträge zur Kentnis des Sporophyten von *Polytrichum juniperinum* Willdenow. *Planta* 14, 244–385.

Wijk, R. van der. (1929). Über den Bau und Entwicklung der Peristomzähne bei *Polytrichum*. *Rec. Trav. Bot. néerl.* 26, 289–395.
